# Effect of Precursor on Antifouling Efficacy of Vertically-Oriented Graphene Nanosheets

**DOI:** 10.3390/nano7070170

**Published:** 2017-07-04

**Authors:** Karthika Prasad, Chaturanga D. Bandara, Shailesh Kumar, Gurinder Pal Singh, Bastian Brockhoff, Kateryna Bazaka, Kostya (Ken) Ostrikov

**Affiliations:** 1 School of Chemistry, Physics and Mechanical Engineering, Queensland University of Technology, Brisbane, QLD 4000, Australia; karthika.prasad@hdr.qut.edu.au (K.P.); chaturangab@yahoo.com (C.D.B.); shailesh.kumar@qut.edu.au (S.K.); gurinderpal.singh@connect.qut.edu.au (G.P.S.); bastian.brockhoff@connect.qut.edu.au (B.B.); kostya.ostrikov@qut.edu.au (K.O.); 2 CSIRO-QUT Joint Sustainable Processes and Devices Laboratory, Commonwealth Scientific and Industrial Research Organisation, P.O. Box 218, Lindfield, NSW 2070, Australia

**Keywords:** graphene, nanowalls, plasma-enabled synthesis

## Abstract

Antifouling efficacy of graphene nanowalls, i.e., substrate-bound vertically-oriented graphene nanosheets, has been demonstrated against biofilm-forming Gram-positive and Gram-negative bacteria. Where graphene nanowalls are typically prepared using costly high-temperature synthesis from high-purity carbon precursors, large-scale applications demand efficient, low-cost processes. The advancement of plasma enabled synthesis techniques in the production of nanomaterials has opened a novel and effective method for converting low-cost natural waste resources to produce nanomaterials with a wide range of applications. Through this work, we report the rapid reforming of sugarcane bagasse, a low-value by-product from sugarcane industry, into high-quality vertically-oriented graphene nanosheets at a relatively low temperature of 400 °C. Electron microscopy showed that graphene nanowalls fabricated from methane were significantly more effective at preventing surface attachment of Gram-negative rod-shaped *Escherichia coli* compared to bagasse-derived graphene, with both surfaces showing antifouling efficacy comparable to copper. Attachment of Gram-positive coccal *Staphylococcus aureus* was lower on the surfaces of both types of graphene compared to that on copper, with bagasse-derived graphene being particularly effective. Toxicity to planktonic bacteria estimated as a reduction in colony-forming units as a result of sample exposure showed that both graphenes effectively retarded cell replication.

## 1. Introduction

Recent times have witnessed a significant increase in the use of nanomaterials, especially graphene, for a wide range of applications, ranging from electronics to agriculture and manufacturing [1,2,3]. However, incorporation of graphene into day-to-day devices demands large-scale production of high-quality graphene which is cost effective [3]. Hence, substantial efforts have been made to develop numerous cost-effective ways of producing high-quality graphene [4,5,6].

The use of natural resources for the production of graphene and graphene-based products has been studied widely [7,8]. Recently, plasma-enhanced chemical vapour deposition (PECVD) has been used for the production of high-quality graphene nanosheets from a variety of resources [9]. In this low-temperature synthesis technique, the graphene can be grown directly on a wide range of desired substrates without any external heating or catalyst, and it is therefore considered a promising method for controllable graphene synthesis [1,3,9,10,11]. Advantageously, vertically-oriented graphene nanosheets, also known as nanowalls, can be fabricated with excellent control over the spatial arrangement, density, and orientation of the nanosheets [12,13]. In addition to this, vertical orientation of surface-immobilised graphene affords the material a number of advantageous properties in comparison with conventional horizontal, randomly oriented graphene sheets, particularly for applications where chemical or biological reactivity and mechanical robustness are desired. The free-standing, self-supported rigid structure of vertically-oriented graphene sheets prevents collapse and/or the random stacking of graphene nanosheets associated with strong van der Waals interactions. Such a mechanically-stable, non-agglomerated morphology ensures high specific surface area (or surface-to-volume ratio), long reactive edges, and abundant open channels between the sheets [14]. These graphene networks can serve as platforms for highly-sensitive and selective field-effect transistor biosensors [15,16].

This work investigates the antibacterial activity of graphene fabricated from raw, multicomponent, low-cost resources, compared to that of graphene derived from high-purity carbon precursor, against pathogenic multi-drug resistant bacterial species, namely Gram-negative *Escherichia coli* and Gram-positive *Staphylococcus aureus*. A fast and reactive plasma-enabled process is used for reforming sugarcane bagasse and methane gas into graphene. This particular graphene growth process does not involve toxic or hazardous gases and does not require any catalyst or external substrate heating to produce thin vertical graphene sheets on the same substrate. This method is also environmentally friendly, does not produce any chemical residue or waste, and is energy and material-efficient.

## 2. Results

### 2.1. Structural and Morphological Characterization of Nanomaterials

**Figure 1:**
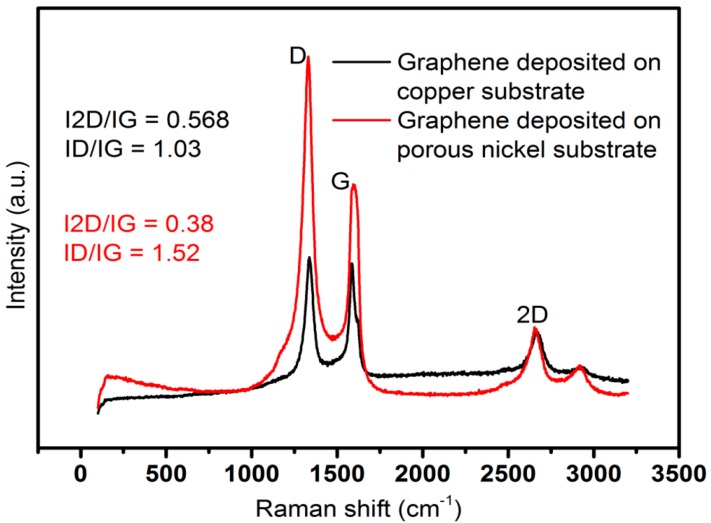
Raman spectra of graphene nanosheets deposited from methane on copper and from bagasse on porous nickel substrates.

Successful formations of graphene from both precursors were confirmed by Raman spectroscopy. The Raman spectra were collected using a Renishaw inVia spectrometer (Renishaw plc, Gloucestershire, United Kingdom) with laser excitations of 633 nm. Figure 1 shows the characteristic bands at 1590 cm^−^^1^ (G band), 1320 cm^−^^1^ (D band) and 2600 cm^−^^1^ (2D), which confirms the formation of graphene from different precursors.

The ratio of intensity of 2D and G bands indicates the number of layers of graphene. The increased ratio indicates the formation of thinner graphene flakes, whereas the ratio is smaller for thicker ones. Here, the thinnest flakes were obtained on plasma-treated copper substrate, where methane was converted to graphene.

The morphology and elemental distribution of the graphene are characterized by Field Emission Scanning Electron Microscopy (FESEM, ZEISS SIGMA VP, Oberkochen, Germany) employing an electron gun voltage of 10 kV with an energy dispersion spectrum (EDS, ZEISS SIGMA , Oberkochen, Germany).

**Figure 2:**
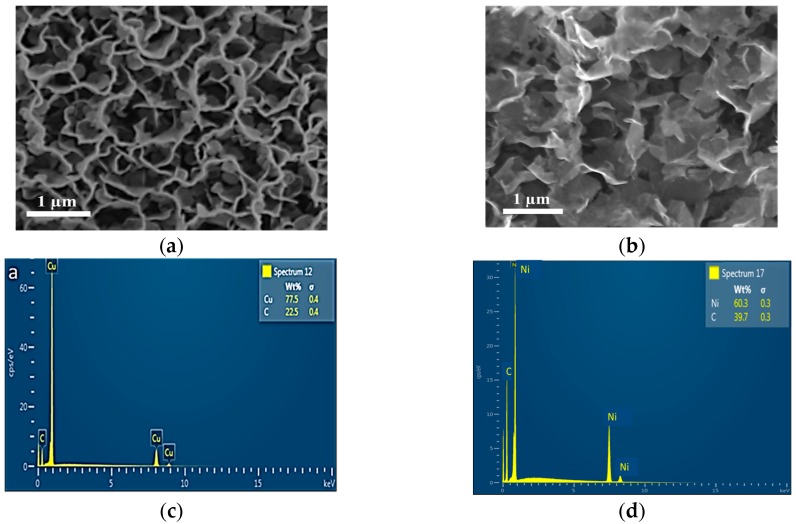
(**a**,**b**). Representative SEM images of graphene nanowalls produced from (**a**) methane on copper substrate and (**b**) from bagasse on nickel substrate. (**c**,**d**) EDS spectra of graphene formed from (**c**) methane on copper substrate and (**d**) from bagasse on nickel substrate.

The SEM images in Figure 2 confirm the findings from Raman spectra. While Figure 2a shows an SEM image of graphene nanosheets deposited on a copper foil using methane as precursor gas, Figure 2b shows the image of graphene nanosheets on a nickel substrate using sugarcane bagasse powder as precursor. SEM images reveal relatively transparent nanosheets on copper foil, which suggests that the deposited nanosheets are thinner than the graphene nanosheets deposited on porous nickel substrate. These results correlate well with the results obtained by calculating the intensity ratios from Raman spectra.

The energy dispersion spectra (EDS) for the samples are shown in Figure 2. The EDS spectra indicate the presence of carbon on copper and porous nickel substrates. The absence of any other additional peaks confirms the contamination-free deposition of graphene nanosheets.

Further characterization of the graphene nanosheets was performed using Transmission Electron Microscopy (TEM). The crystal images of graphene nanosheets were collected using JOEL 2100F HR-TEM (JOEL USA, Peabody, MA, USA). The electron beam energy used for this analysis was 200 keV.

**Figure 3:**
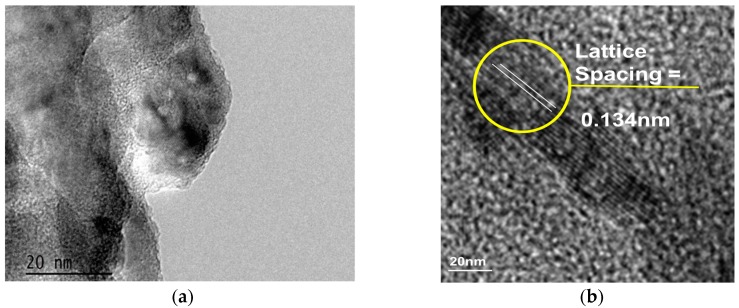
TEM images of the samples deposited from (**a**) methane on copper substrate, and (**b**) bagasse on porous nickel substrate.

TEM samples were prepared by placing a drop of graphene dispersed in isopropanol on a carbon-coated copper grid and subsequently evaporating the isopropanol. Figure 3 represents the TEM images of graphene nanosheets deposited from methane and sugarcane bagasse on copper and nickel substrates, respectively. Images clearly show the graphitic edges and the crystalline structure of deposited graphene. On nickel substrate, the formation of thicker graphene is evident from the greater number of layers formed. The interplanar distance between subsequent two layers was 0.134 nm. With an average number of layers between 6 and 9, vertically-oriented methane-derived graphene nanowalls are notably thinner than that of bagasse-derived graphene, with the latter typically having between 15 and 20 layers.

### 2.2. Antibacterial Studies

**Figure 4:**
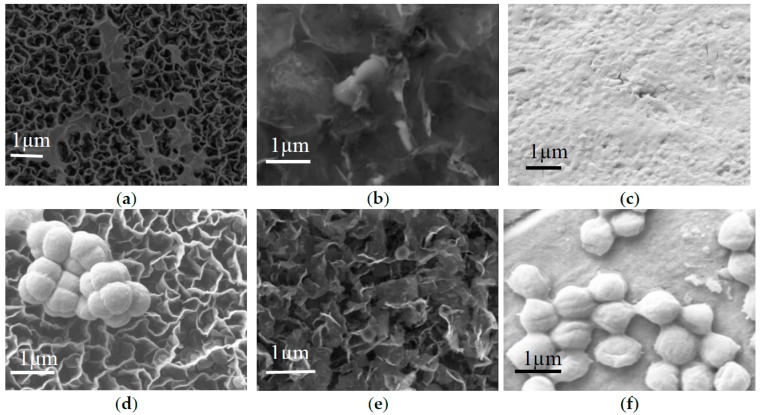
Representative SEM images of *E. coli* cell attachment on the surfaces of (**a**) methane-derived (GNW_M) and (**b**) bagasse-derived (GNW_B) graphene, and (**c**) pure copper substrate after 4 h of incubation at 22 °C. SEM images of *S. aureus* cell attachment on the surfaces of (**d**) GNW_M, (**e**) GNW_B, and (**f**) copper after incubation under the same conditions.

The antibacterial efficacy of two different graphene samples against Gram-negative rod-shaped *E.* *coli* and Gram-positive coccal *S.* *aureus* bacteria were investigated in terms of cell attachment and toxicity to planktonic cells. For this purpose, cell cultures were incubated in the presence of different graphene samples and copper in 30 µL of Luria broth at room temperature. After 4 h of incubation, the surfaces of the samples were visualised using SEM. As evident from Figure 4, graphene nanowalls fabricated from methane were significantly more effective in preventing surface attachment of *E. coli* compared to bagasse-derived graphene, with both surfaces showing antifouling efficacy comparable to copper. Attachment of *S. aureus* was lower on the surfaces of both types of graphene compared to that on copper, with bagasse-derived graphene being particularly effective.

**Figure 5:**
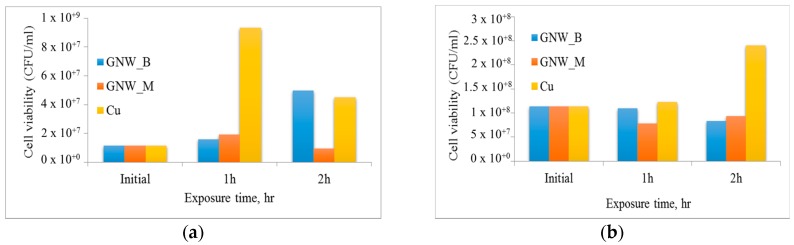
The survival rate of (**a**) *E. coli* and (**b**) *S. aureus* bacteria when exposed to graphene fabricated from methane (GNW_M) and bagasse (GNW_B), and a pure copper substrate.

Toxicity of the surfaces to planktonic bacteria was investigated by estimating the number of colony-forming units (i.e., live cells) at different times during the incubation period. The results of this study are summarized in Figure 5. Taking into consideration the growth rate of *E. coli* bacteria, graphene samples fabricated from bagasse (GNW_B) showed considerable toxicity against planktonic bacteria. Although the cell numbers gradually increased over the period of incubation, the cell numbers were below (at 1 h) or similar to (at 2 h) those on copper. On the other hand, graphene derived from methane (GNW_M) effectively retarded cell replication, with cell numbers increasing only slightly during the first hour of exposure, and then deceasing to below the initial seed values. The efficacy of methane-derived graphene was significantly better than that of copper, a known broad-spectrum antibacterial agent. Cells incubated in the presence of copper surfaces first experienced limited antibacterial action from copper, with cell numbers reaching 9.3 × 10^7^ CFU/mL. However, after 2 h of exposure, there was a significant reduction in the number of viable cells, attributed to the diffusion of copper ions from the surface of the substrate.

In contrast to *E. coli* bacteria, the growth of *S. aureus* was effectively retarded by graphene nanowalls from bagasse (GNW_B) and methane (GNW_M), with the latter being characterized by slightly lower numbers of surviving organisms at 2 h. The growth of cells incubated in the presence of copper was limited in the first hour of incubation. However, the cell numbers increased significantly after 2 h of incubation, and were substantially higher than those observed for cells incubated in the presence of graphene samples, reaching approximately 9.6 × 10^8^ CFU/mL.

As a broad-spectrum antifouling and antibacterial agent, GNW_M is more efficient than GNW_B for the pathogens tested, i.e., *E. coli* and *S. aureus.*

## 3. Discussion

In this experiment, we successfully synthesised graphene using methane gas and sugarcane bagasse as the precursors. The Raman spectra of the obtained graphene (Figure 1) shows the characteristic graphene bands with G band at 1590 cm^−^^1^, D band at 1320 cm^−^^1^ and 2D band at 2600 cm^−^^1^ (2D). This confirms the formation of a graphitic structure [17]. Moreover, from the intensity ratio of 2D and G bands, which indicates the number of layers of graphene, it is evident that the thinnest flakes were obtained on plasma-treated copper substrate, where methane was converted to graphene. Further, the D and G bands give an insight about the crystallinity of the structure, which can be determined by calculating the ratio of intensities of D and G bands (ID/IG). With a decrease in the value of ID/IG, the crystallinity of the structure increases [10]. Therefore, in this experiment, the ratio of ID to IG was lower for graphene formed from methane gas, which shows that the graphene formed was highly crystalline.

The high-resolution SEM images in Figure 2 clearly show the formation of vertically-aligned graphene nanosheets on both substrates. The images correlated well with the findings from the intensity ratio of Raman spectra and show formation of thinner graphene layer on copper substrate and thicker ones on nickel substrate. The absence of any other peak in the EDS spectra (Figure 2) demonstrates the purity of the graphene formed. The graphitic edges and the crystalline structure of deposited graphene are shown in TEM images in Figure 3.

The morphology of graphene layers grown by means of plasma-enhanced synthesis has been previously shown to depend on the chemistry and state of the precursor, as well as on the properties of the catalyst. The growth of vertical graphene is considered to be a step-flow process on the basis of nucleation at the bottom, and the thickness of graphene will depend on the number of layers that nucleates from the bottom [11].Transition metals, such as nickel and copper, are commonly used for graphene production due to their ability to readily absorb and interact with carbon sources due to their partially filled d sub-shell [18]. When compared with nickel, copper exhibits comparatively low carbon solubility, which leads to a different mechanism of graphene formation. Specifically, the growth of graphene on copper is dominated by the direct deposition of carbon atoms on the catalyst surface, with limited diffusion of carbon atoms into the copper, which enables growth of thinner graphene layers [19]. On the other hand, when nickel is used as a catalyst, carbon atoms from the carbonaceous source diffuse readily into the catalyst bulk during the high-temperature processing, and precipitate to the catalyst surface during the cooling period. Since the growth mechanism combines that of surface growth and precipitation of carbon from the catalyst bulk, the structure of resulting graphene may differ substantially from that deposited on Cu substrate [20,21]. This was evident from the characterization performed on graphene produced in this study. 

The antifouling efficacy of methane- and bagasse-derived graphene surfaces was comparable to or better than that of copper. There are several possible mechanisms that may be responsible for the bactericidal activity of graphene [22]. The observed differences in antifouling and bactericidal activity of graphene against phenotypically-distinct bacteria, namely *E*. *coli* and *S*. *aureus*, shown in Figure 4 and Figure 5 can be at least in part attributed to differences in physico-chemical properties of these materials. The sharp edges characteristic of vertically-oriented graphenes are one of the most important mechanisms in terms of antibacterial activity [23], where the sharp edges of graphene may physically disrupt cellular membranes, resulting in the loss of bacterial membrane integrity, which may lead to leakage of intracellular substances, and eventually to cell death [23]. Sharper edges of methane-derived graphene may thus be more effective in compromising the integrity of the soft membrane of *E.* *coli*, resulting in contact bacterial inactivation on the surface [24].

Attachment of *S. aureus* was lower on the surfaces of both types of graphene compared to that on copper, with bagasse-derived graphene being particularly effective. The electron transfer mechanism from a microbial membrane to graphene is another mechanism for the destruction of bacteria [25], which may be particularly relevant in this case. Graphene-based materials induce oxidative stress towards the endogenous antioxidant glutathione. Here, the graphene acts as a conductor between the negatively charged cell and the metal. This electron flow towards the graphene metal substrate ultimately results in cell death [26].

In addition to mechanisms associated with direct cell-surface contact, oxidative stress induced by graphene nanowalls may inhibit the bacterial metabolism and disrupt essential cellular functions, eventually leading to cellular inactivation. Oxidative stress can include reactive oxygen species (ROS)-dependent or ROS-independent pathways. In the former, the stress is induced by excess accumulation of extracellular ROS species, such as hydrogen peroxide, superoxide anions, hydroxyl radicals, and singlet molecular oxygen. These ROS species induce lipid peroxidation, intercellular protein inactivation and gradual disintegration of cell membrane, followed by the eventual cell death. ROS-independent oxidative stress oxidizes the vital cellular structure without ROS production, which can be induced by the charge transfer from cellular membrane to graphene, where graphene acts as an electron pump [27,28].

In comparison to this, the antifouling mechanism of copper involves several processes. At the initial stages, non-enzymatic peroxidation of membrane phospholipids takes place, leading to loss of membrane integrity. This may be followed by rapid and extensive degradation of genomic DNA and necrotic cell death. The time of onset of killing, the rate of cell death, and the kinetics of lipid peroxidation are inherently linked to structural characteristics and metabolic state of the cell. Furthermore, it is possible that *S. aureus* is more efficient that *E. coli* in terms of surface attachment and production of extracellular polymeric substances on the surface of copper, which may mask the surface and prevent the process of contact-mediated membrane peroxidation. This is supported by the SEM images (Figure 4), which show significantly higher numbers of attached *S. aureus* cells on the copper surface. In contrast, the surface copper substrate exposed to *E. coli* cells remains minimally colonised [29].

Our results indicate that graphene nanowalls from methane and bagasse are as efficient as copper in preventing surface colonization by bacterial strains tested, attributed to thin, sharp edges of the thus-produced graphene, as well as the ability of graphene walls to transfer elections and induce oxidative stress on the cell. Although methane-derived graphene is slightly more effective against *E. coli*, the lower-cost bagasse-derived graphene provides attractive antifouling and antibacterial activity for large-scale applications.

## 4. Materials and Methods

The deposition of vertically-oriented graphene nanosheeets was carried out in a RF inductively coupled plasma CVD system. A polycrystalline copper film was used as a substrate for the graphene to grow from methane gas precursor, whereas a 99%-pure porous nickel foam was used for the reforming of sugarcane bagasse. For each deposition, 0.5 mg of sugarcane bagasse was placed evenly on the substrate prior to being loaded into the camber. A low-temperature inductively coupled plasma was used to both reform sugarcane bagasse, to heat the polycrystalline copper catalyst, and to dissociate the hydrocarbon gas precursor. 

**Figure 6:**
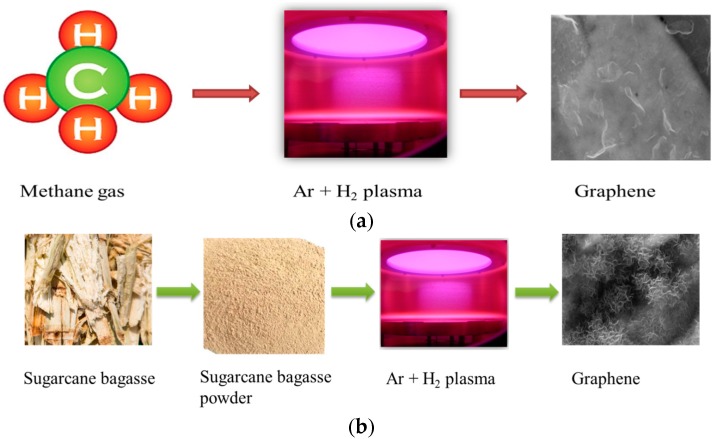
Schematic representation of plasma enabled synthesis of graphene. (**a**) Conversion of methane gas into graphene in the presence of plasma. (**b**) Reforming of sugarcane bagasse into graphene.

In Figure 6a, the CH_4_ precursor was heavily diluted in hydrogen (H_2_) and argon (Ar). Growth is carried out over a range of plasma exposure times, with best results obtained at 10 min. A gas mixture of H_2_/Ar/CH_4_ at a flow rate of 85/10/5 sccm, respectively, was fed into the chamber for deposition.

Figure 6b represents the formation of graphene nanosheets through reforming sugarcane bagasse with plasma-enabled synthesis. Powdered sugarcane bagasse was evenly distributed on the surface of the porous nickel substrate. A gas mixture of H_2_/Ar was fed into the chamber at a flow rate of 50 and 15 sccm, respectively. In both the experiments, the chamber pressure was maintained at 2.0 Pa and plasma was generated using RF power of 760 W and the deposition was carried out for 10 min.

For the qualitative analysis of bactericidal activity of graphene against *E.* *coli* and *S.* *aureus*, bacterial cultures were refreshed on nutrient agar plates from a stock culture and grown overnight at 37 °C in a 5 mL of nutrient broth [30]. The culture was collected at the logarithmic stage of growth and washed twice with 0.01 M PBS solution (Sigma Aldrich, St. Louis, MO, USA) (pH = 7.4). An aliquot of 1 mL of bacterial suspension from an adjusted OD_600_ = 0.1 bacterial suspension was placed on the surface of graphene nanowalls on fabricated on copper and nickel substrates and was allowed to incubate for 4 h at room temperature (22 °C) in a cell culture plate. Untreated copper and nickel substrate was used as control. After every 1 h, a 10 μL aliquot was taken and a 10× dilution series (10^−^^1^ to 10^−^^8^) was made, and from the resulting 100 μL solution, 30 μL was plated on nutrient agar media, in triplicate, for each solution. Plates were incubated overnight at 37 °C, and colonies for each aliquot between 3 and 30 were counted and recorded with their respective dilution factor. This experiment was conducted in triplicate.

## 5. Conclusions

**Figure 7:**
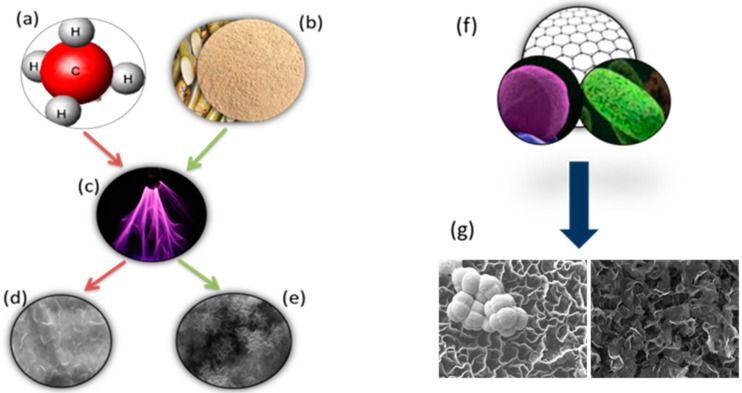
Comparative evaluation of the antibacterial efficacy of graphene nanowalls synthesised from different precursors. High-cost, high-purity conventional, i.e., methane (**a**), and low-cost, natural carbon, i.e., sugarcane bagasse (**b**) sources are converted using low-temperature plasma process (**c**) into high-quality graphene sheets (**d**,**e**). When bacterial cells are exposed to thus-fabricated surface-immobilised graphenes (**f**), anti-bacterial activity of graphene is observed to differ (**g**), expressed in terms of cell attachment and number of colony-forming units.

Production of high-quality, large-area graphene sheets in a cost-effective way has always been a challenge [31,32]. Low-temperature plasma-enabled processing has recently emerged as a highly-versatile family of techniques for controlled synthesis of nanomaterials [33,34] and modification of abiotic [35] and biological objects [36,37]. In this paper, we have successfully demonstrated a cost-effective, single-step plasma-enabled synthesis of graphene from methane gas and sugarcane bagasse (Figure 7). Methane gas and sugarcane bagasse were completely transformed into high-quality graphene within 10 min at 400 °C. We also demonstrated the antifouling efficiency of the thus-produced graphene, and concluded that the graphene synthesised from the methane gas was more crystalline, thinner and more effective against bacteria.
